# Endocrine Function in Thalassemia Intermedia

**Published:** 2006-09

**Authors:** H. Karamifar, M. Karimi, G. H. Amirhakimi, M. Badiei

**Affiliations:** 1 *Division of Endocrinology and Metabolism, Department of Pediatrics, Shiraz University of Medical Sciences, Shiraz, Iran;*; 2 *Hematology Research Center, Department of Pediatrics, Shiraz University of Medical Sciences, Shiraz, Iran*

**Keywords:** thalassemia intermedia, endocrinopathies

## Abstract

Thalassemias are the most common genetic disorder on a wordwide basis. β-thalassemia is a severe hemolytic anemia which results from genetic defects in the synthesis of the hemoglobin β-chain. Various endocrine abnormalities have been described in patients with thalassemia major. Endocrine disturbances have also been observed in patients with thalassemia intermedia (TI). In this study endocrine functions were investigated in TI and here the frequency of different abnormalities is reported. Ninety-three patients (40 males, 53 females) with thalassemia intermedia, 11-40 years old (mean 19.4 yr) were studied. Medical history was obtained and a complete physical examination was done for each patient. The age, sex, weight, height and serum ferritin levels were recorded using a questionnaire. Growth Hormone (GH) secretion, thyrotropin (TSH), T^4^, parathyroid hormone (PTH) and cortisole levels were determined in these patients. The mean ± Standard Deviation (SD) serum ferritin level was 452.4 ± 312.60 μg/L. Mean ± SD hemoglobin concentration was 9 ± 1 g/dl. Short stature was present in 46% of patients. Growth hormone deficiency was one of the most frequent (31%) endocrine abnormalities in these patients. Primary hypothyroidism was observed in 21.5% of patients. Hypoparathyroidism was found in one patient (1%). Failure of puberty was present in 2% of patients, secondary ammenorrhea was observed in 6.4% of patients and diabetes mellitus (DM) in 2% of patients. Conclusion: Growth retardation and GH deficiency should be considered as common finding in TI. Therefore endocrine evaluation of these patients is suggested to prevent complications and to improve the overall quality of life.

## INTRODUCTION

Thalassemias are the most common genetic disorder on a worldwide basis (1), β-thalassemia is a severe hemolytic anemia which result from genetic defects in the synthesis of the hemoglobin β chain (2). Thalassemia intermedia is a medical condition in which individuals have inherited an affected β-gene from both the mother and father (i.e they are homozygous for β-thalassemia) but they demonstrate milder clinical symptoms than patients with thalassaemia major. Individuals with TI manage to maintain hemoglobin levels between 6-9 g/dl and may not require regular blood transfusions (3).

Various endocrine abnormalities have been described in patients with thalassemia major and β-thalassemia intermedia (4, 5) and most reports suggest iron overload as a possible factor in the development of target-organ dysfunction (4, 6). Patients with thalassemia major, frequently present endocrine complications mainly due to organ damage secondary to iron overload (7). Patients with TI are either not transfused or are transfused less frequently than patients with thalassemia major. Hence their iron stores are lower than in thalassemia major varying from mildly to moderately increased (8) and as a consequence in TI one would expect less severe endocrine abnormalities and in a smaller percentage of patients according to the degree of iron overload. There are no studies addressing the prevalence of endocrine dysfunctions in patients with TI in Iran. The aim of this study was to evaluate of endocrine functions in patients with TI in Iran.

## PATIENTS AND METHODS

Ninety-three patients (40 males and 53 females) with thalassernia intermedia, aged 11-40 years old (mean age 19.45 ± 5.40 yr) were selected out of 220 TI patients randomly in this study. Medical history was obtained and a complete physical examination was done for each patient. Age, sex, weight and height, age of initiation of blood transfusion and age of initiation of desferrioxamine, its regular or irregular administration were recorded using a questionnaire. Height was measured by a single observer using a stadiometer. Pubertal staging was performed by the same observer and was assessed according to Tanner staging (9) Lack of pubertal development was indicated by absence of testes enlargement in boys over 14 years and no breast development in girls over 13 years. Secondary amenorrhea was defined as the absence of periods for 6 months or longer after menstrual cyclicity has been established. Liver function tests, serum albumin, serum calcium (Ca^++^), phosphorus and blood sugar were measured. Blood samples were taken at least 2 weeks after the last blood transfusion. Serum ferritin level was determined by Elisa method. Serum cortisol and T^4^ were measured by Radioimmune Assay (RIA) standard commercial kit (Kavoshiar, Iran) and TSH by Immunoradiometric Assay (IRMA) with standard commercial kit (Kavoshiar, Iran). The GH secretion was determined after provocative L-Dopa tests with levodopa (500 mg/1.73m^2^) primed by propranolol 0.75 mg/kg, maximum 40 mg orally. Blood samples were taken at 0 (baseline), 30, 60, 90 and 120 minutes after an overnight fasting and GH was measured by IRMA with standard commercial kit (Orion Diagnostic, Finland). Girls and boys over 11 and 13 years of age received ethinyl estradiol (0.02 mg) and methyl testosterone 10 mg respectively three times a day for 2 days before L-Dopa test. Growth hormone deficiency was diagnosed if GH level in all sample was below 10 ng/ml.

Serum PTH was determined in those patients whose serum Ca^++^ level was below 8 mg/dl and phosphate above 6.5 mg/dl by IRMA commertial kit (Biosurce Europe S.A.).

Hypoparathyroidism was diagnosed if PTH level was below 13 Pg/ml (normal range 13-66 pg/ml) in patients with a serum Ca^++^ below 8 mg/dl and phosphorus over 6.5 mg/d1.

Primary hypothyroidism was diagnosed if T^4^ was < 4.5 μg/dl (normal range 4.5 - 12 μg/dl) and TSII >4 mlU/1 (0.3-3.8 mIU/1). Compensated hypothyroidism (subclinical hypothyroidism) defined as an elevated serum TSH level but normal concentration of T^4^ (10). Secondary hypothyroidism when T^4^ was <4.5 μg/dl and TSH was normal or decreased.

Serum cortisol was measured at 8 Am after overnight fasting. Adrenal insufficiency: serum cortisol <10 μg/dl and clinical manifestation.

Diabetes Mellitus was diagnosed if fasting blood sugar ≥126 mg/dl on 2 separate occasions the study was approved by the institutional review board. For data analysis student’s t-test, Chi-square test, Fisher exact test and Pearson’s correlation coefficient test were performed. P value less than 0.05 was considered significant. The results are presented as mean±SD.

## RESULTS

A total of 93 β-thalassemia intermedia patients were studied. The age of the patients ranged from 11-40 years (mean 19.4 ± 5.40). Characteristics of patients data are summerized in Table 1. Fifty-six patients had 12 blood transfusions per year during their disease and 37 patients below 3 transfusion/year (without transfusion). Desferrioxamine injection was regularly given in 16% of patients and irregularly used in 84% of the patients. The mean serum ferritin level in patients with multiple blood transfusions was 546 ± 343 μg/l and in patients without transfusions was 310.80 ± 188.80 μg/l. this difference was statistically significant (*P*<0.001).

**Table 1:** Characteristics of TI patients

Number of patients	93

Male/female	40 (43%)/53 (57%)
Mean age start blood transfusion	5.8 ± 3.5
Mean hemoglobin	9 ± 1 g/dl
Mean serum calcium	9.5 ± 0.6 mg/dl
Mean serum phosphorous	5 ± 0.9 mg/dl
Mean weight	43.6 ± 9 kg
Mean height	155.5 ± 11cm
Short-stature	46%
Mean age start desferrioxamine (years)	
All cases	9.6 ± 4.9
GH deficient	10.7 ± 4.7
GH Sufficient	9 ± 5 (*P*=0.3)
Hypothyroidism	7.5 ± 4.5
Euthyroidism	10.3 ± 4.9 (*P*=0.08)
DM	7.5 ± 5
Without DM	9.7 ± 5 (*P*=0.5)
Mean serum ferritin levels (µg/l)	
All cases	452.4 ± 312.6
GH deficient	463 ± 300
GH Sufficient	447.7 ± 320 (*P*=0.8)
Hypothyroidism	509.6 ± 378.5
Euthyroidism	436.8 ± 293 (*P*=0.4)
DM	558.5 ± 4.5
Without DM	450 ± 312.6 (*P*=0.6)

### Serum albumin was in normal range in all patients

Short stature was found in 46% of patients defined by height below the 3rd percentile for age. Short stature was found in 43% of patients with multiple transfusions and 51% of patients without transfusions. This difference was not statistically significant (*P*=0.9). There was no significant difference between height of patients with GH deficient and GH sufficient (*P*=0.3).

Table 2 shows the endocrine functions of the TI patients. Growth hormone deficiency was present in 31% of the patients. (26% female, 37.5% male). The mean GH was 7.50 ± 4 ng/ml. Growth hormone deficiency in patients with blood transfusion was 35% and in patients without blood transfusion was 29% (*P*=0.6).

**Table 2:** Endocrine functions of 93 patients with thalassemia intermedia

Variable	Primary hypothyroidism	Diabetes deficiency	GH defieiency	Secondary amenorrhea

All cases (No. %)	20 (21.5)	2 (2)	29 (31)	6 (6.4)
Mean ± SD age (years)	19 ± 4	21 ± 0	21 ± 5	23.7 ± 4
Mean ± SD age at start of desferrioxamine therapy (years)	7.5 ± 4.5	7.5 ± 5	10.7 ± 4.7	14.5 ± 2
Mean ± SD serum ferritin level (µg/l)	509.6 ± 378.5	558.5 ± 415	463 ± 300.8	503.7 ± 284

The mean age of patients with GH sufficiency was 18.7 ± 5.5 years and in patients with GH deficiency was 21 ± 4.9 years, the difference was statistically significant (*P*=0.03).

Compensated hypothyroidism was present in 19% of patients and primary hypothyroidism in 2% of patients. Hypothyroidism in patients with blood transfusion was 22% and in patients without transfusion was 21% and the difference was not significant statistically (*P*=1).

Hypoparathyroidism was present in only one patient (1%) (serum calcium 7.1 mg/dl, phosphorus 11 mg/dl), who had history of blood transfusion. No adrenal insufficiency was found in these patients. The mean serum cortisol was 15.8 ± 4.8μg/dl.

Diabetes mellitus was found in 2% of patients with the mean age of 21 years. Diabetes mellitus in patients with blood transfusion was 3.6% and there was no DM in patients without blood transfusion (*P*=0.5).

Failure of puberty was present in 2% of the patients. Secondary amenorrhea was present in 6.4% of the patients. The mean serum ferritin level in patients with secondary amenorrhea was 503.6 ± 284 μg/l and in patients without amenorrhea was 393.5 ± 287.2 μg/l (*P*=0.3).

Table 1. Fifty-six patients had 12 blood transfusions per year during their disease and 37 patients below 3 transfusion/year (without transfusion). Desferrioxamine injection was regularly given in 16% of patients and irregularly used in 84% of the patients. The mean serum ferritin level in patients with multiple blood transfusions was 546 ± 343 μg/l and in patients without transfusions was 310.80 ± 188.80 μg/l. this difference was statistically significant (*P*<0.001).

Table 2 shows the endocrine functions of the TI patients. Growth hormone deficiency was present in 31% of the patients. (26% female, 37.5% male). The mean GH was 7.50 ± 4 ng/ml. Growth hormone deficiency in patients with blood transfusion was 35% and in patients without blood transfusion was 29% (*P*=0.6).

## DISCUSSIONS

This study was designed to evaluate the endocrine complications in patients with TI from Iran. Iron overload and secondary hemochromatosis have been described in several types of hemolytic anemias including TI (5, 6). Thalassemia intermedia patients do not present serious growth problem and they generally reach adult age without or with little requirement of blood transfusion (11). But like thalassemia major they may present clinical problems of iron overload. It has been shown that iron overload in thalassemic patients results from increased intestinal iron absorption rather than external iron load (blood transfusion) (12).

Short stature was common in our study, height more than 2 SD below man height for age (below 3rd percentile). De Sanctis *et al*. (13) reported the prevalence of short stature in 7% of patients. The difference may be due to poor contorl, irregular follow-up, under nutrition and low economic status in our patients. The mechanism of short-stature in these patients seems to be multifactorial. Growth retardation has been attributed to GH deficiency, hypothyroidism, diabetes mellitus, zinc deficiency and low Hb levels.

Growth Hormone deficiency was common in the present report. There were no relationship between GH deficiency and serum ferritin level. To the best of our knowledge there is no study about GH deficiency in TI patients. Growth hormone deficiency seems to be a frequent complication of TI in patients treated according to current protocols. Forty-one percent of the patients had at least one endocrine dysfunction and there were no relationship between endocrine dysfunction and serum ferritin level, age of start of desferrioxamine and hemoglubin level. De Sanctis (13) reported that appearance of endocrine complications was not necessarily related to the degree of iron overload as evaluated by the serum ferritin. Among their patients with endocrine complications some had ferritin levels lower than 100 μg/l. Striking elevations of hepatic iron concentration have been observed in patients with TI with only slight increases in serum ferritin levels.

In the study of De-Sanctis, hypothyroidism detected in 5.7% of patients. The difference in incidence of hypothyroidism noted is probably related to differences in transfusion.

None of the De-Sanctis patients had hypoparathyroidism. The prevalence of delayed puberty in the present series was very lower than those reported (13).

Lack of concordance of ferritin concentrations and endocrine dysfunction is in part due to the fact that serum ferritin levels increase linearly with the transfusion load up to 100 units of transfused blood, but thereafter there is no simple relationship. Misleading ferritin levels also occur with chronic inflammatory liver diseases (14). There is no direct relationship between the amount of iron accumulated and organ dysfunction, it is possible that endocrine glands are extremely sensitive to iron toxicity and that even small amounts of it accumulated in the first years of life produce damage that can not be reversed (15).

Abnormal glucose tolerance test (OGTT) was found in 24% of patients in De Sanctis study (13). The thalassemics with impaired OGTT had higher serum ferritin levels and lower Hb levels than those with normal OGTT. In a study by E-Flatau (2) abnormal glucose tolerance was detected in 2 out of 4 patients in these 2 patients serum ferritin levels were more than 20 times the normal value. In our study there were no relationship between DM and serum ferritin levels, Hb and age of start of desferrioxamine therapy. It is suggested that factors like family history genetic background, malnutrition and hepatitis have a role in the pathogenesis of DM in this group of patients.

We concluded that growth retardation and GH deficiency should be considered as common finding in TI. Therefore endocrine evaluation of these patients is suggested to prevent complications and to improve the overall quality of life.
